# Immune checkpoint inhibitor-induced cardiotoxicity in patients with lung cancer: a systematic review and meta-analysis

**DOI:** 10.1186/s40959-024-00229-x

**Published:** 2024-06-17

**Authors:** Naser Yamani, Aymen Ahmed, Gabriel Ruiz, Amraha Zubair, Fariha Arif, Farouk Mookadam

**Affiliations:** 1 Division of Cardiology, Banner University Medical Center, University of Arizona, Phoenix, AZ USA; 2 Department of Medicine, Dow University of Health Sciences, Karachi, Pakistan

**Keywords:** Immune checkpoint inhibitor, Cardiotoxicity, Cardiac adverse event, Chemotherapy, Lung cancer, Arrythmia, Heart failure

## Abstract

**Background:**

The use of immune checkpoint inhibitors (ICIs) for the treatment of lung cancer may precipitate cardiotoxic events. We aimed to perform a meta-analysis to evaluate the cardiotoxicity associated with ICIs in patients with lung cancer.

**Methods:**

A literature search was conducted across four electronic databases (Cochrane CENTRAL, MEDLINE, OVID EMBASE and Google Scholar) from inception through 31st May 2023. Randomized controlled trials (RCTs) assessing the impact of ICIs on cardiac outcomes in lung cancer patients were considered for inclusion. Risk ratios (RR) with 95% confidence intervals (CIs) were pooled and analysis was performed using a random-effects model. The Grading of Recommendations Assessment, Development and Evaluation approach was followed to assess confidence in the estimates of effect (i.e., the quality of evidence).

**Results:**

A total of 30 studies including 16,331 patients, were included in the analysis. Pooled results showed that single ICI (RR: 2.15; 95% CI: 1.13–4.12; *p* = 0.02; I2 = 0%) or a combination of single ICI plus chemotherapy (RR: 1.38 [1.05–1.82]; *p* = 0.02) significantly increased the risk of cardiac adverse events when compared with chemotherapy alone. No significant difference was noted when a dual ICI (RR: 0.48 [0.13–1.80]; *p* = 0.27) was compared with single ICI. In addition, there was no significant association between the use of ICIs and incidence of cardiac failure (RR: 1.11 [0.48–2.58]; *p* = 0.80), or arrhythmia (RR: 1.87; [0.69–5.08]; *p* = 0.22).

**Conclusion:**

Compared with chemotherapy alone, use of a single ICI or a combination of single ICI plus chemotherapy significantly increased the risk of cardiotoxicity. However, employing dual immunotherapy did not result in a significant increase in the risk of cardiotoxicity when compared to the use of a single ICI.

**Supplementary Information:**

The online version contains supplementary material available at 10.1186/s40959-024-00229-x.

## Background

Lung cancer is a leading cause of mortality and morbidity, claiming around 127,000 lives (21% of all cancer fatalities) in the United States annually [1]. Immune Checkpoint Inhibitors (ICIs) are increasingly being used for the treatment of lung cancer and have been shown to improve clinical outcomes, including overall survival and progression-free survival [2]. Guidelines have been established to direct the appropriate use of ICIs for the treatment of lung cancer. The National Comprehensive Cancer Network (NCCN) guidelines advocate the use of ICIs as a first-line therapy for patients with advanced non-small cell lung cancer (NSCLC) [3]. The United States Food and Drug Administration has also approved several ICIs including tremelimumab, nivolumab, atezolizumab, avelumab, ipilimumab, pembrolizumab, and durvalumab [4]. While the benefits reaped with ICIs play a pivotal role in the treatment of lung cancer, recent studies have shown that ICIs may precipitate serious cardiotoxic events, such as myocarditis, pericarditis, arrhythmias, myocardial infarction (MI), and non-inflammatory left ventricular dysfunction [5–10]. Concern over poor cardiac outcomes is heightened by the extensive use of ICIs in lung cancer therapy [5]. NCCN recommendations have also recognized the potential cardiotoxicity associated with ICIs and advised vigilant monitoring and management of cardiovascular adverse events [3]. Although some meta-analyses have been conducted, current evidence regarding the cardiotoxicity of ICIs in patients with lung cancer remains largely inconclusive due to the inclusion of patients with different types of cancers and evaluation of limited types of ICIs in prior studies. Given the conflicting findings in recently published studies and the paucity of data related to cardiotoxicity assessment in patients with lung cancer, we decided to conduct a meta-analysis to evaluate the cardiotoxic effects of ICIs in patients with lung cancer (small cell lung cancer (SCLC) and NSCLC), when used in isolation, in conjunction with other ICIs or in conjunction with standard chemotherapy.

## Methods

This systematic review and meta-analysis has been reported in concordance with guidelines provided by preferred reporting items for systematic review and meta-analyses (PRISMA) [11]. Approval from the institutional review board was not required since the data was publicly available.

### Data sources and search strategy

An electronic search of Cochrane CENTRAL, MEDLINE, OVID EMBASE and Google Scholar databases was conducted for Randomized controlled trials (RCTs) assessing the cardiotoxic effects associated with the use of ICIs in lung cancer patients, from their inception through 31st May 2023, without any time or language restrictions. Search strategy for each database which is provided in Supplementary Table 1. Moreover, we used generic, trade and pharmaceutical names of all ICIs to search for additional published trials on clinicaltrials.gov. In addition, we manually screened the reference list of retrieved trials, previous meta-analyses and review articles to identify any relevant studies.

### Study selection and data extraction

All articles retrieved from the systematic search were exported to EndNote Reference Manager (Version X7.5; Clarivate Analytics, Philadelphia, Pennsylvania, 2016) where duplicates were sought and removed. The remaining articles were then assessed at title and abstract level by two independent investigators (AZ and FA), after which full text were read to confirm relevance. Any disagreements were resolved by mutual discussion with a third investigator (AA). Studies were included if they (a) were published RCTs with a follow-up duration of at least 24 weeks; (b) included adult male or female (≥ 18 years of age) patients with lung cancer; (c) compared ICIs with placebo/chemotherapy/dual ICI; and (d) reported at least one cardiotoxic outcome. Single arm and observational studies were not considered.

### Data extraction and outcomes of interest

Two investigators (AZ and FA) autonomously extracted data from the selected studies on pre-specified collection forms. Data were extracted, including first author, publication year, study ID, study design, trial phase, treatments, sample size in each arm, tumor type and stage, follow-up time, outcome measures. The primary outcome of this meta-analysis was the presence of any adverse cardiac event between the treatment and control arms (Single-ICI vs. Chemotherapy, Single-ICI + Chemotherapy vs. Chemotherapy, and Single-ICI vs. Dual-ICl). The secondary outcomes were the incidence of cardiac adverse events following the use of ICI-related therapy such as myocarditis, arrhythmia, MI, cardiac failure and atrial fibrillation, when compared with control group. Risks of bias were assessed independently using the Cochrane Risk of Bias Tool [12]. 

### Statistical analysis

The statistical analysis was performed by extracting Risk Ratios (RR) and their corresponding 95% Confidence Intervals (CIs) from each trial, focusing on cardiotoxicity events. More precisely, the RR was computed by extracting dichotomous outcomes as the number of participants who experienced an event and the total number of participants in each arm of the trial. Data was pooled using the generic inverse variance method and random-effects model in the Cochrane Review Manager software (RevMan version 5.4.1). Forest plots were created to assess visually the results of pooling. Heterogeneity across studies was evaluated using Higgins *I*^2^ and a value less than 50% for *I*^2^ was considered acceptable, while 50–75% indicates substantial heterogeneity, and greater than 75% indicates significant heterogeneity [13]. Sub-group analyses were performed based on the two types of lung cancer, SCLC and NSCLC and different combinations of chemotherapy. Additionally, we performed leave-one-out sensitivity analysis to evaluate if any single study disproportionately influenced the results of the primary outcome. A visual inspection of the funnel plot was conducted to assess the publication bias. To assess the confidence in the estimates of effect (i.e., quality of evidence) across studies, we followed the Grading of Recommendations Assessment, Development and Evaluation GRADE approach by making judgments about the risk of bias, publication bias, indirectness, imprecision, and inconsistency among different trials [14]. A *p*-value < 0.05 was considered significant in all cases.

## Results

### Literature search

The initial literature search yielded 16,693 potentially relevant articles. After applying the pre-determined eligibility criteria, 30 studies (encompassing 31 trials) were included in this meta-analysis [2, 15–43]. The PRISMA flowchart summarizes the results of our literature search (Supplementary Fig. 1) [11].

### Study characteristics and quality assessment

Our short-listed studies included 16,331 patients (70.2% males; mean age 63.35 years) over a median follow-up of 18.1 months. Ten of these were phase 2 studies, while 23 were phase 3 studies. Nine studies included patients with SCLC, while 22 trials included NSCLC. The characteristics of each study are shown in Table 1. Quality assessment showed an overall low risk of bias among studies (Supplementary Figs. 2 and 3). Visual inspection of funnel plot did not reveal any small study or publication bias. (Supplementary Fig. 4)

**Table 1:** Characteristics of included studies

First Author	Year	Study ID	Study Design	Trial Phase	Tumor Type	Treatment	Sample Size	Median Follow Up (month)
Cheng Y^14^	2022	ASTRUM-005	international, double-blind, randomized	3	extensive stage SCLC	Serplulimab plus chemotherapy vs. placebo plus chemotherapy	585	12.3
Zhou C^15^	2022	GEMSTONE-302	randomised, double-blind	3	stage IV squamous or non-squamous NSCLC	Sugemalimab plus chemotherapy vs. Placebo plus chemotherapy	479	8.6
Wang J^16^	2022	CAPSTONE-1	multicentre, randomised, double-blind,	3	extensive stage SCLC	Adebrelimab plus chemotherapy vs. placebo plus chemotherapy	462	13.5
Taniguchi Y^17^	2022	TORG1630 jRCTs031180331	multi-institutional, open-label	2/3	NSCLC stage IIIB/ IIIC/IV, received one or two previous chemotherapy regimens	Nivolumab vs. nivolumab plus docetaxel	129	-
Brien MO^18^	2022	KEYNOTE-091	randomised, triple-blind	3	stage IB, II, or IIIA NSCLC with previous adjuvant chemotherapy	Pembrolizumab vs. placebo	1171	35.6
Altorki^20^	2021	NCT02904954	single-centre, open-label, randomised, controlled	2	clinical stages I-IIIA NSCLC	Neoadjuvant durvalumab alone versus neoadjuvant durvalumab + stereotactic radiotherapy	60	16.9
Antonia^19^	2016	NCT01928394	multicentre, open-label	1/2	limited-stage or extensive-stage SCLC, had disease progression after at least one previous platinum-containing regimen	Nivolumab versus Nivolumab + ipilimumab	213	6.6
Boyer^21^	2021	KEYNOTE-598	randomized, double-blind	3	Metastatic NSCLC PDL1 tumor proportion score > = 50%	Pembrolizumab versus Pembrolizumab + ipilimumab	568	24.0
Gettinger^22^	2021	Lung-MAP S1400I	open-label randomized	3	previously treated patients with Stage IV squamous Cell Lung Cancer	Nivolumab + ipilimumab versus Nivolumab	246	29.5
Jotte^23^	2020	IMpower131	global, open-label	3	stage IV squamous NSCLC	Atezolizumab + carboplatin + paclitaxel versus atezolizumab + carboplatin + nabpaclitaxel versus carboplatin + nab-paclitaxel	1000	18.1
Langer^24^	2016	KEYNOTE-021	randomised, open-label	2	advanced NSCLC	Pembrolizumab + chemotherapy versus Chemotherapy	123	10.6
Malhotra^25^	2021	NCT03026166	multicenter, open-label	1–2	Previously Treated Extensive-Stage SCLC	Rova-T + nivolumab versus Rova-T + nivolumab and ilimumab	42	7.3
Mazieres^26^	2021	POPLAR	randomized, open-label	2	previously treated advanced NSCLC	Atezolizumab versus Docetaxel	277	48.0
Mazieres^26^	2021	OAK	randomized, open-label	3	previously treated advanced NSCLC	Atezolizumab versus Docetaxel	1187	48.0
Mok^27^	2019	KEYNOTE-042	multicenter, randomized, open-label	3	previously untreated, PD-L1-expressing, locally advanced or metastatic NSCLC	Pembrolizumab versus Chemotherapy	1274	12.8
Nishio^28^	2021	IMpower132	multicenter, randomized, openlabel	3	advanced NSCLC	Atezolizumab + Chemotherapy versus Chemotherapy	101	17.5
Pakkala^29^	2020	NCT02701400	randomized, two-arm, non-comparative	2	relapsed SCLC	Durvalumab(D) + tremelimumab(T) without SBRT versus SBRT followed D/T	18	5.7
Rodríguez-Abreu^30^	2021	KEYNOTE-189	double-blind trial	3	metastatic nonsquamous NSCLC without sensitizing EGFR/ ALK alterations	Pembrolizumab + chemotherapy versus Placebo + chemotherapy	607	31.0
Schoenfeld^31^	2022	NCT02888743	open-label, multicentre, randomised	2	metastatic NSCLC refractory to previous PD(L)-1 therapy	Durvalumab–tremelimumab ± radiotherapy	78	12.4
Sezer^32^	2021	EMPOWER-Lung 1	multicentre, open-label, global	3	advanced NSCLC	Cemiplimab versus Chemotherapy	697	13.1
Welsh^33^	2020	NCT02444741	prospective randomized	1/2	metastatic NSCLC	Pembrolizumab with or without radiation therapy	100	20.4
Antonia^34^	2017	PACIFIC	randomized, double-blind, international	3	stage III, locally advanced	Durvalumab versus placebo	709	14.5
Barlesi^35^	2018	JAVELIN Lung 200	open-label, multicentre, randomised	3	stage IIIB, IV, or recurrent NSCLC with disease progression after previous platinum doublet treatment	Avelumab group versus Docetaxel	758	18.9
Borghaei^36^	2015	CheckMate-057	randomized, open-label, international	3	stage IIIB or IV or recurrent nonsquamous NSCLC after radiation therapy or surgical resection	Nivolumab versus Docetaxel	555	-
Herbst^2^	2016	KEYNOTE-010	open-label, multicentre, randomised	2/3	previously treated, PD-L1-positive, advanced NSCLC	Pembrolizumab versus Pembrolizumab versus Docetaxel	991	13.1
Horn^37^	2018	IMpower133	double-blind, placebo-controlled,	3	Extensive-Stage SCLC	Atezolizumab versus Placebo	394	13.9
Paz-Ares^38^	2019	CASPIAN	open-label, multicentre, randomised	3	extensive-stage SCLC	Durvalumab + platinum– etoposide versus Platinum–etoposide	531	14.2
Reck^42^	2016	CA184-156	multicenter, randomized, double-blind	3	Extensive-Stage SCLC	Chemotherapy/Ipilimumab versus Chemotherapy/Placebo	954	10.5
Socinski^39^	2018	Impower-150	international, randomised, open-label	3	Metastatic Nonsquamous NSCLC who had not previously received chemotherapy	atezolizumab + BCP versus bevacizumab + carboplatin + paclitaxel (BCP)	787	15.5
West^41^	2019	Impower-130	multicentre, randomised, open-label	3	metastatic NSCLC	Atezolizumab + chemotherapy versus Chemotherapy	705	19.2
Carbone^40^	2017	CheckMate-026	multicentre, randomised, open-label	3	Stage IV or Recurrent NSCLC	Nivolumab versus Chemotherapy	530	13.5

### Primary outcomes

#### Single ICI versus chemotherapy

**Fig. 1:**
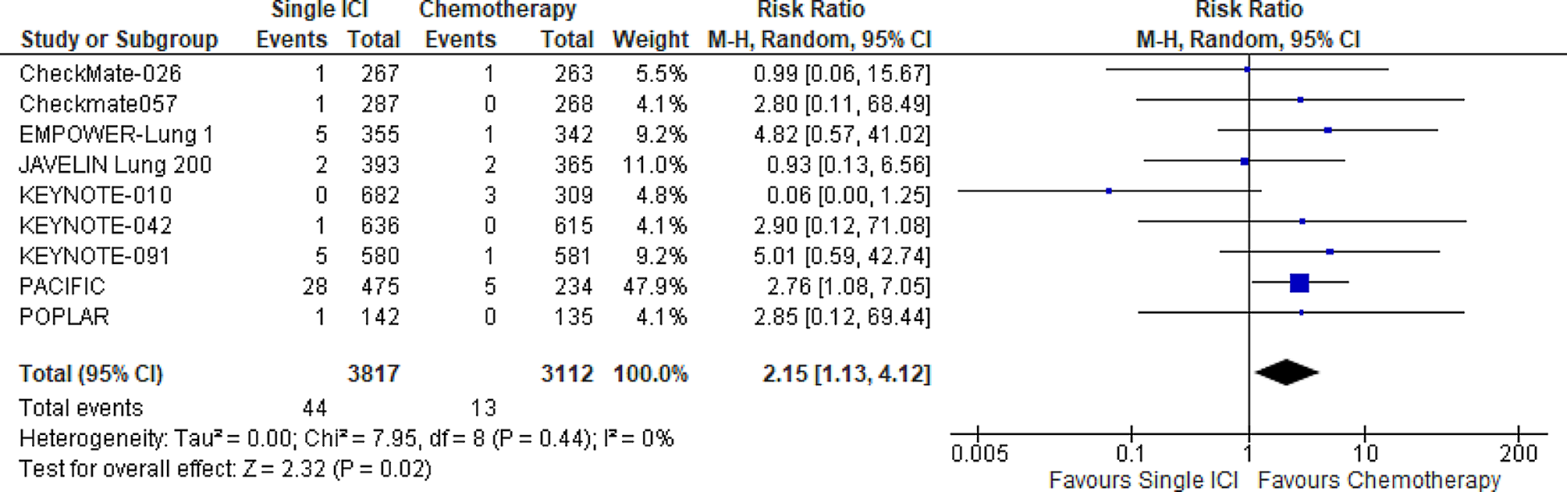
Forest plot of risk ratio of any cardiac adverse events among patients with lung cancer, Single immune checkpoint inhibitor vs. Chemotherapy

Treatment with single ICI (*n* = 9 trials, 6,929 patients) significantly increased the risk of any cardiac adverse events when compared with chemotherapy (RR: 2.15; 95% CI: 1.13–4.12; *p* = 0.02; I^2^ = 0%). (Fig. 1) Our results stayed consistent upon sensitivity analysis. Overall, the quality of evidence was graded low. (Supplementary Table 2)

#### Single ICI plus chemotherapy versus chemotherapy

**Fig. 2:**
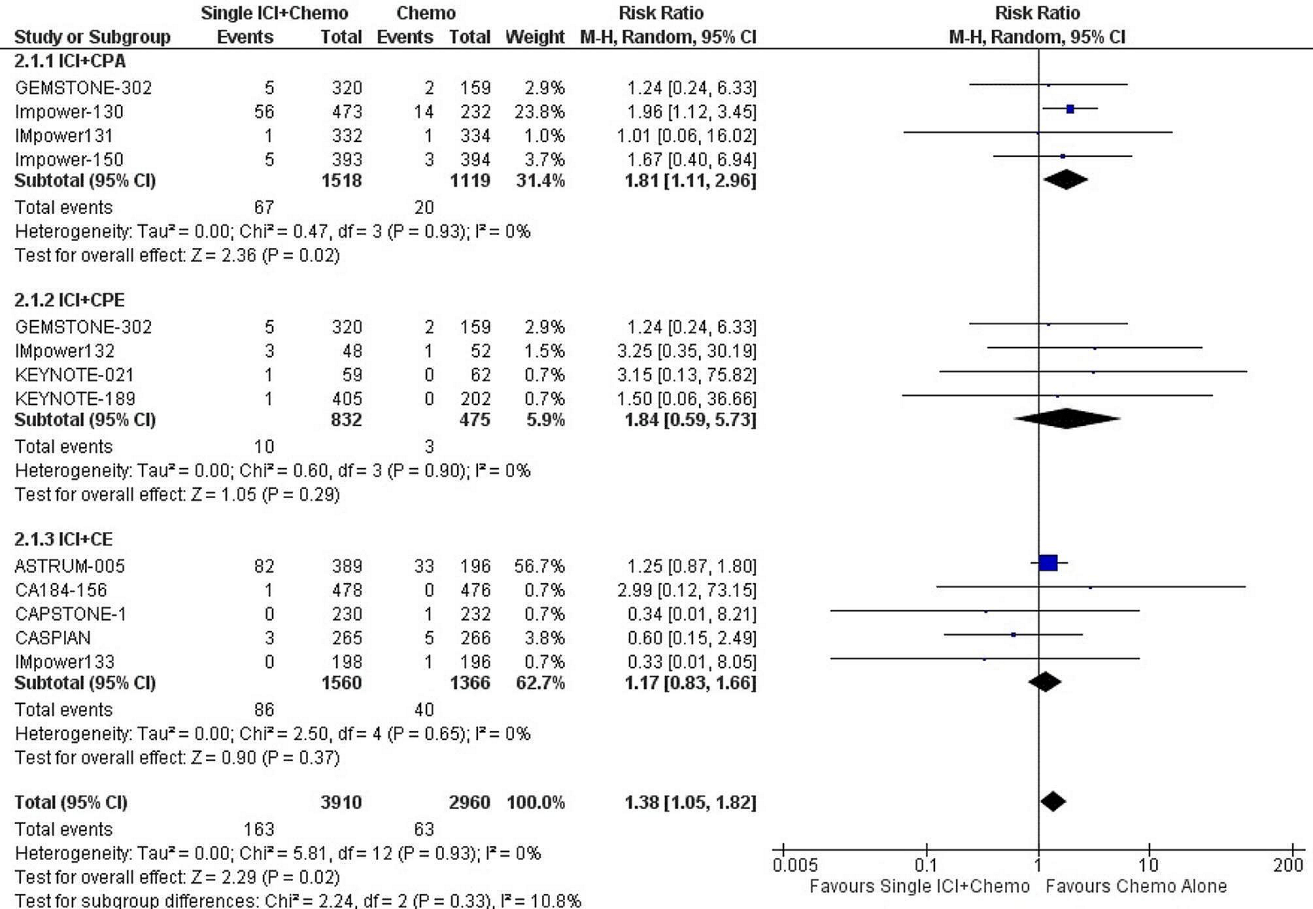
Forest plot of risk ratio of any cardiac adverse events among patients with lung cancer, Single immune checkpoint inhibitor + Chemotherapy vs. Chemotherapy

Treatment with single ICI plus chemotherapy (*n* = 12 trials, 6,391 patients) significantly increased the risk of any cardiac adverse events when compared with chemotherapy (RR: 1.38; 95% CI: 1.05–1.82; *p* = 0.02; I^2^ = 0%). (Fig. 2) Our results were consistent upon sensitivity analysis. Overall, the quality of evidence was graded high. (Supplementary Table 2)

#### Single ICI versus dual ICI

**Fig. 3:**
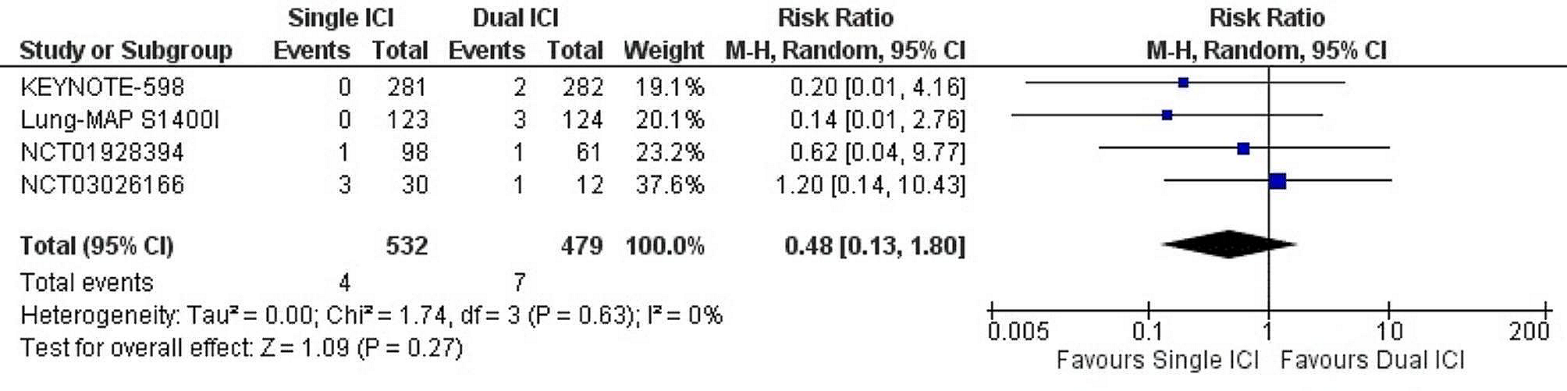
Forest plot of risk ratio of any cardiac adverse events among patients with lung cancer, Single immune checkpoint inhibitor vs. Dual immune checkpoint inhibitors

Treatment with single ICI (*n* = 4 trials, 1,011 patients) did not significantly decrease the risk of any cardiac adverse events when compared with dual ICI (RR: 0.48; 95% CI: 0.13–1.80; *p* = 0.27; I^2^ = 0%). (Fig. 3) Our results were consistent upon sensitivity analysis. Overall, the quality of evidence was graded low. (Supplementary Table 2)

### Secondary outcomes

#### Cardiac failure

**Fig. 4:**
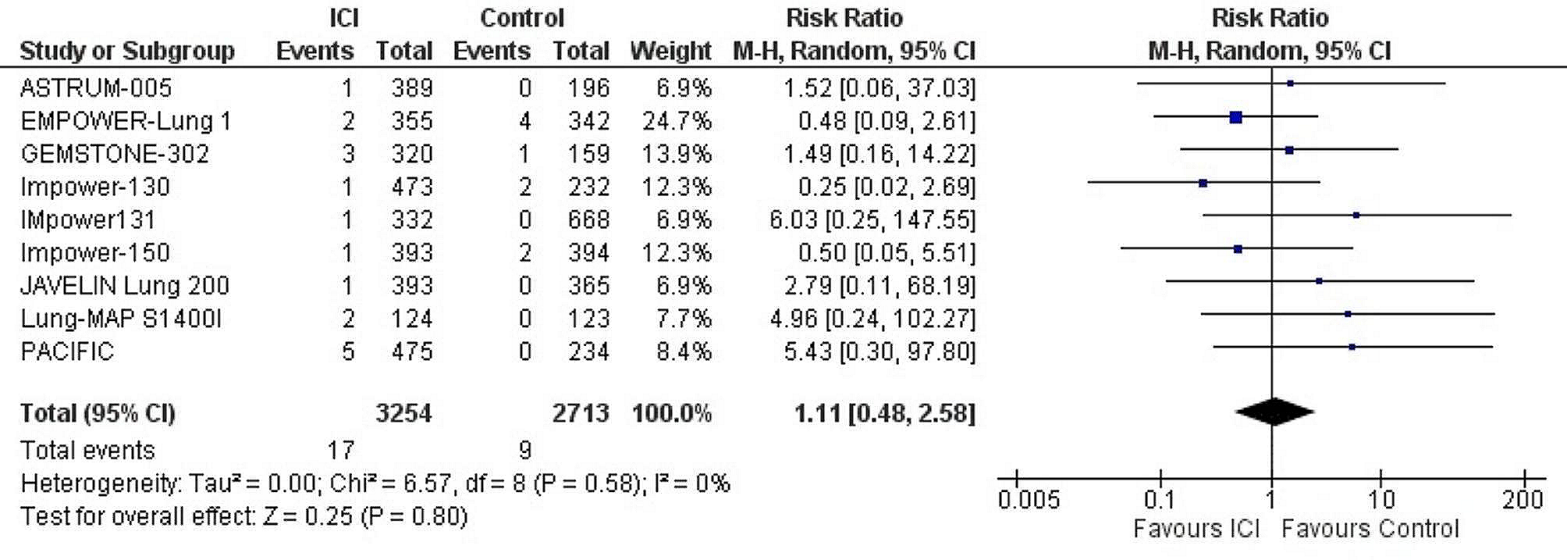
Forest plot of risk ratio of cardiac failure in lung cancer patients treated with immune checkpoint inhibitors vs. control

The use of ICI had no significant effect on the occurrence of cardiac failure, when compared with control group (placebo or chemotherapy). (*n* = 9 studies, 5,574 patients) (RR: 1.11; 95% CI: 0.48–2.58; *p* = 0.80; I^2^ = 0%). (Fig. 4)

#### Myocarditis

**Fig. 5:**
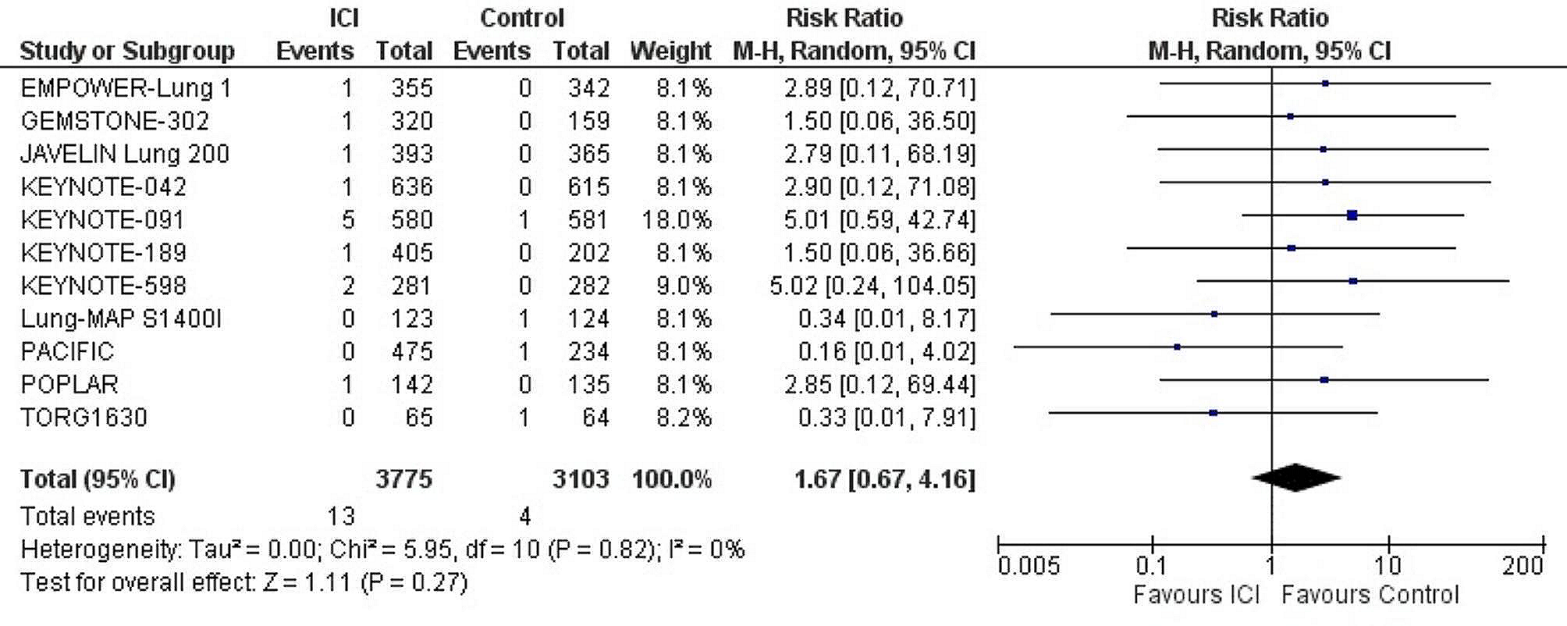
Forest plot of risk ratio of myocarditis in lung cancer patients treated with immune checkpoint inhibitors vs. control

ICIs had no significant effect on the incidence of myocarditis when compared with control group (placebo or chemotherapy). (*n* = 11 studies, 6,878 patients) (RR: 1.67; 95% CI: 0.67–4.16; *p* = 0.27; I^2^ = 0%). (Fig. 5)

#### Arrhythmia

ICIs did not significantly increase the risk of arrhythmia when compared with control (chemotherapy or placebo) (*n* = 5 studies, 2,591 patients) (RR: 1.87; 95% CI: 0.69–5.08; *p* = 0.22; I^2^ = 16%). Supplementary Fig. 5.

#### Myocardial infarction

ICIs did not significantly increase the risk of MI when compared with control (chemotherapy or placebo) (*n* = 8 studies, 4,726 patients) (RR: 1.23; 95% CI: 0.48–3.18; *p* = 0.66; I^2^ = 0%). Supplementary Fig. 6.

#### Atrial fibrillation

ICIs did not significantly increase the risk of atrial fibrillation when compared with control, however there appeared to be a trend (chemotherapy or placebo) (*n* = 7 studies, 3,535 patients) (RR: 1.19; 95% CI: 0.40–3.55; *p* = 0.76; I^2^ = 24%). Supplementary Fig. 7.

### Subgroup analyses

On subgroup analysis by type of chemotherapy, ICI with carboplatin and paclitaxel (CPA) significantly increased the risk of cardiotoxicity compared with CPA (*n* = 4 studies, 2,637 patients) (RR: 1.81; 95% CI: 1.11–2.96; *p* = 0.02; I^2^ = 0%). No significant difference in cardiotoxicity was reported between ICI with carboplatin and pemetrexed (CPE) and CPE (*n* = 4 studies, 1,307 patients) (RR: 1.84; 95% CI: 0.59–5.73; *p* = 0.29; I^2^ = 0%) and ICI with carboplatin and etoposide (CE) and CE (*n* = 9 studies, 2,926 patients) (RR: 1.17; 95% CI: 0.83–1.66; *p* = 0.37; I^2^ = 0%). (Fig. 2)

In addition, subgroup analysis by type of lung cancer revealed that ICI-administered patients with NSCLC (*n* = 22 studies, 11,911 patients) reported a significantly higher risk of cardiac adverse events (RR: 1.79; 95% CI: 1.24–2.60; *p* = 0.002; I2 = 0%) while patients with SCLC (*n* = 9 studies, 3,932 patients) did not have any significant risk of cardiac adverse events (RR: 1.16; 95% CI: 0.84–1.61; *p* = 0.37; I2 = 0%) (P-interaction = 0.09). (Supplementary Fig. 8)

## Discussion

Our meta-analysis including over 16,000 patients outlines various key findings. First, patients treated with either single ICI or a combination of single ICI plus chemotherapy exhibited significantly higher rates of cardiotoxicity, when compared with chemotherapy alone. Second, single ICI alone did not precipitate any significant risk of cardiotoxic events when compared with dual ICIs. Third, no significant association was found between the use of ICIs and the incidences of cardiac failure, cardiac arrhythmia, myocarditis, MI, and atrial fibrillation when compared with control group.

Our findings concur with a prior meta-analysis conducted by *Zhang et al.*. on lung cancer patients which demonstrated that there was no significantly increased risk of cardiotoxicity with dual ICI vs. single ICI groups [44]. However, in contrast with our findings, a recent network meta-analysis by *Jin et al.* showed that the administration of a single ICI (CTLA-4) plus chemotherapy compared with dual ICI therapy did not give rise to any significant cardiotoxic effects [45]. Other meta-analyses evaluating the cardiotoxicity of ICIs have also revealed conflicting findings [46, 47] However, these meta-analyses were not specific to patients with lung cancer and included patients with different types of malignancies. Our meta-analysis, based on a larger sample size, specifically evaluates the cardiotoxicity associated with the use of ICIs in patients with lung cancer.

The risk of cardiotoxicity significantly increased with single ICI therapy when compared with chemotherapy. This finding is supported by *Salem et al.*, who conducted an analysis using data from cancer patients treated with ICI therapy sourced from Vigibase (The World Health Organization’s international database of case safety reports) and identified that the treatment with ICI monotherapy reported cardiac adverse events including myocarditis, pericardial diseases and temporal arteritis [48]. The prevailing hypothesis regarding the pathophysiology of ICI-induced cardiotoxicity suggests that ICI inhibit certain muscle specific antigens such as against troponin, myosin or desmin, that are commonly shared between the tumor cells and cardiomyocytes, triggering a cross-reactive response with T cells targeting both the tumor and the cardiac muscle, resulting in immune related adverse events [49]. Moreover, it’s noteworthy that the acute myocarditis that may occur with ICIs can be fatal and fulminant if not recognized early and managed appropriately [50]. Hence, patients using PD-1/PD-L1/CTLA-4 inhibitors should undergo routine clinic monitoring of heart function, including cardiac troponin, electrocardiogram (ECG), cardiac ultrasonography.

In addition, our results suggest that combination ICI plus chemotherapy poses a greater risk of cardiotoxicity compared with ICI monotherapy. A possible reason is that the ICI-accompanied impairment of immune regulation mechanisms and potential synergistic action of chemotherapy-related inflammation could lead to an overwhelming inflammatory response that may prove detrimental to the heart [51]. Our findings concur with a prior study by *Zhang et al.* which found that adding ICIs to chemotherapy increased the risk of cardiotoxicity by 67% compared with chemotherapy alone [44]. Similarly, a 7.3% incidence of cardiac disorders was observed in patients < 75 years receiving the combination of atezolizumab plus chemotherapy in the *IMpower132* trial [29]. The intensified cardiotoxic risk when these medications are administered together demands careful patient monitoring and a thorough assessment of heart health. When prescribing the combination regimen, clinicians should proceed with caution and consider the possibility of enhanced cardiotoxicity. Further investigation is needed to determine whether combination therapy increases the risk of serious cardiotoxicity (≥ grade III). This underscores the critical significance of customized patient evaluation and tailored treatment methods in proactively managing and mitigating the increased risk of cardiac adverse events associated with the concomitant use of ICIs and chemotherapy. Moreover, the direct cardiotoxic action of some chemotherapeutic drugs, such as anthracyclines, may be amplified by ICIs [6]. Although an analysis conducted by *Rohit Bishnoi et al.* based on the Surveillance, Epidemiology, and End Results Program (SEER) database found a lower incidence of cardiotoxicity with combination ICI plus chemotherapy, the study was retrospective in nature and liable to inherent biases which may have modified the cardiac outcomes [52]. 

The use of dual ICIs did not significantly increase the risk of cardiotoxicity compared with a single ICI. Dual ICI therapy for lung cancer patients has demonstrated a manageable safety profile with no appreciable increase in the risk of cardiotoxicity [53]. The beneficial outcomes could be attributed to the synergistic effects of dual ICIs, which promote T-cell-mediated immune responses, thereby increasing anticancer activity, while maintaining cardiac safety [54]. *Puzanov et al.* found no significant difference in the incidence of myocarditis between dual ICI and single ICI groups, suggesting that the cardiotoxicity does not substantially increase with the addition of a second ICI [55]. 

Although there was no significant association with the use of ICIs and individual cardiac adverse events, our analysis, in alignment with the prior meta-analysis by *Zhang et al.* (incidence rate ratio: 0.014), revealed that cardiac arrhythmia was the most predominant adverse cardiac event associated with the usage of ICIs (RR: 1.87) [44]. Immunotherapy-induced arrhythmias have an uncertain underlying mechanism that has not yet been fully elucidated. Hence, it is important to monitor clinical symptoms, ECG and biomarkers till further evidence is available. On the other hand, while *Zhang et al.* reported that myocarditis is the least occurring cardiac adverse event (Incidence rate ratio: 0.003), our meta-analysis identified it as the second most frequent cardiac adverse event (RR: 1.67). Given that PD-L1, PD-1, and CTLA-4 play significant roles in the communication between the immune system and the heart, myocarditis has known immune linked etiology. Disruption of these pathways can result in autoimmune myocarditis and subsequent heart failure.

The number of patients exposed to ICIs is anticipated to rise significantly due to over 40 approved indications for their use and the possibility of new indications in the future [4]. This can potentially worsen the risk of fatal cardiac outcomes in patients with lung cancer. Refractory arrhythmias with ICI-associated myocarditis are the major cause of fatalities [5]. Fatal cardiac events are often noted earlier than non-fatal events due to the underlying cardiac inflammation that may rapidly unleash with the initiation of ICIs [47]. Moreover, decreased functional reserve predisposes the elderly population to detrimental events, and opportunistic infections due to long-term immunosuppression can complicate the clinical course [56]. Patients at high risk have a poor prognosis and must be monitored closely, with reassessment of immunotherapy if symptoms appear. Clinicians should take important predisposing factors such as age, concomitant medications, baseline cardiac function, and cardiac history into account when initiating ICI therapy especially in patients at low risk. Finding new risk factors and biomarkers is essential for preventing the incidence of cardiotoxicity as the number of patients continues to rise.

Some limitations must be kept in mind while interpreting the results of our study. Firstly, the inclusion of distinct lung cancer types (SCLC and NSCLC) across multiple stages (I to IV) and diverse chemotherapeutic drugs and dosing regimens (single ICI, dual ICI, ICI combined with chemotherapy, chemotherapy alone, placebo, and radiotherapy) leads to substantial heterogeneity. While subgroup analyses were performed to overcome these differences, the diverse study characteristics remain a limitation, affecting overall generalizability and uniformity of our findings. Secondly, variations in follow-up durations and sample sizes may have affected our results. Thirdly, this is a study-level meta-analysis since individual patient data were not available. Lastly, we may underestimate the risk of cardiac toxicity since some clinical trials only provided data for severe cardiovascular events.

## Conclusion

In conclusion, the administration of a single ICI or a combination of single ICI plus chemotherapy led to an increased risk of cardiotoxicity when compared with chemotherapy. However, our results showed that dual immunotherapy did not have a higher risk of cardiotoxicity when compared with single ICI. Well-powered RCTs with longer follow up durations are required in future to confirm the current evidence of cardiotoxicity associated with ICIs.

### Electronic supplementary material

Below is the link to the electronic supplementary material.

Supplementary Material 1

## Data Availability

All data generated or analyzed during this study are included in this published article and its Supplementary Appendix.

## Abbreviations

ICIs: Immune checkpoint inhibitors
RCTs: Randomized clinical trials
MI: Myocardial infarction
RRs: Risk ratios
SCLC: Small-cell lung cancer
NSCLC: Non-small cell lung cancer
