# Supplementary material for: Immune checkpoint inhibitor-induced cardiotoxicity in patients with lung cancer: a systematic review and meta-analysis
Source: Cardiooncology. 2024 Jun 17;10:37. doi: 10.1186/s40959-024-00229-x (PMC11181582; doi:10.1186/s40959-024-00229-x)

**Supplementary Appendix**

**Supplementary Table 1** Search terms on PubMed

**Supplementary Table 2** Quality of Evidence by GRADE approach

**Supplementary Figure 1** Flowchart of Study selection

**Supplementary Figure 2** Risk of bias graph: review authors' judgements about each risk of bias item presented as percentages across all included studies.

**Supplementary Figure 3** Risk of bias summary: review authors' judgements about each risk of bias item for each included study

**Supplementary Figure 4** Funnel plot of incidence of any cardiac adverse events in lung cancer patients treated with immune checkpoint inhibitors

**Supplementary Figure 5** Forest plot of risk ratio of arrhythmia in lung cancer patients treated with immune checkpoint inhibitors vs control

**Supplementary Figure 6** Forest plot of risk ratio of myocardial infarction in lung cancer patients treated with immune checkpoint inhibitors vs control

**Supplementary Figure 7** Forest plot of risk ratio of atrial fibrillation in lung cancer patients treated with immune checkpoint inhibitors vs control

**Supplementary Figure 8** Forest plot of incidence of any cardiac adverse events in lung cancer patients treated with immune checkpoint inhibitors

**Supplementary Table 1:** Search strategy

| **Database (Articles Retrieved)** | **Search Strategy** |
| --- | --- |
| **MEDLINE**  (5,601 results) | (('lung cancer'[Title/Abstract]) OR ('NSCLC'[Title/Abstract]) OR ('SCLC'[Title/Abstract])) AND (('Atezolizumab' [Title/Abstract]) OR ('Avelumab' [Title/Abstract]) OR ('Nivolumab'[Title/Abstract]) OR ('BMS936559' [Title/Abstract]) OR ('BMS-936559' [Title/Abstract]) OR ('Durvalumab' [Title/Abstract]) OR ('Ipilimumab' [Title/Abstract]) OR ('Pembrolizumab'[Title/Abstract])OR ('Pidilizumab' [Title/Abstract]) OR ('Tremelimumab' [Title/Abstract]) OR ('Spartalizumab' [Title/Abstract]) OR ('Cemiplimab' [Title/Abstract])). (Cardiotoxicity OR "adverse cardiac effects" OR "adverse cardiac events" OR "cardiac toxicity" OR "myocardial toxicity" OR "Cardiotoxicity"[Mesh]) OR ("Immune Checkpoint Inhibitors/adverse effects"[Mesh] OR "Immune Checkpoint Inhibitors/toxicity"[Mesh] OR "Immune Checkpoint Inhibitors"[Mesh]) AND (nivolumab OR Opdivo OR Ipilimumab OR Yervoy OR atezolizumab OR avelumab OR durvalumab OR pembrolizumab OR Cemiplimab OR keytruda OR "anti-programmed cell death-1 monoclonal antibodies" OR "Anti-PD-1 monoclonal antibodies" OR "PD-1" OR "PD-L1" OR "CTLA-4") AND (“lung cancer” OR “lung neoplasm”) |
| (285 results) | ("lung neoplasms"[MeSH Terms] OR ("lung"[All Fields] AND "neoplasms"[All Fields]) OR "lung neoplasms"[All Fields] OR ("lung"[All Fields] AND "cancer"[All Fields]) OR "lung cancer"[All Fields] OR ("lung neoplasms"[MeSH Terms] OR ("lung"[All Fields] AND "neoplasms"[All Fields]) OR "lung neoplasms"[All Fields] OR ("lung"[All Fields] AND "neoplasm"[All Fields]) OR "lung neoplasm"[All Fields]) OR "sclc"[All Fields] OR ("carcinoma, non small cell lung"[MeSH Terms] OR ("carcinoma"[All Fields] AND "non small cell"[All Fields] AND "lung"[All Fields]) OR "non-small-cell lung carcinoma"[All Fields] OR "nsclc"[All Fields] OR "nsclc s"[All Fields] OR "nsclcs"[All Fields])) AND ("immune checkpoint inhibitors"[Pharmacological Action] OR "immune checkpoint inhibitors"[MeSH Terms] OR ("immune"[All Fields] AND "checkpoint"[All Fields] AND "inhibitors"[All Fields]) OR "immune checkpoint inhibitors"[All Fields] OR ("immune"[All Fields] AND "checkpoint"[All Fields] AND "inhibitor"[All Fields]) OR "immune checkpoint inhibitor"[All Fields] OR "ICI"[All Fields]) AND ("cardiotoxic"[All Fields] OR "cardiotoxicity"[MeSH Terms] OR "cardiotoxicity"[All Fields] OR "cardiotoxicities"[All Fields] OR "cardiotoxity"[All Fields] OR ("heart failure"[MeSH Terms] OR ("heart"[All Fields] AND "failure"[All Fields]) OR "heart failure"[All Fields]) OR ("myocardic"[All Fields] OR "myocarditis"[MeSH Terms] OR "myocarditis"[All Fields] OR "myocarditides"[All Fields]) OR ("arrhythmias, cardiac"[MeSH Terms] OR ("arrhythmias"[All Fields] AND "cardiac"[All Fields]) OR "cardiac arrhythmias"[All Fields] OR "arrythmia"[All Fields] OR "arrythmias"[All Fields]) OR ("myocardial infarction"[MeSH Terms] OR ("myocardial"[All Fields] AND "infarction"[All Fields]) OR "myocardial infarction"[All Fields]) OR (("cardiacs"[All Fields] OR "heart"[MeSH Terms] OR "heart"[All Fields] OR "cardiac"[All Fields]) AND ("adverse"[All Fields] OR "adversely"[All Fields] OR "adverses"[All Fields]) AND ("event"[All Fields] OR "event s"[All Fields] OR "events"[All Fields]))) |
| **Google Scholar**  (9,790 results) | (Lung cancer OR lung neoplasm) AND (immune checkpoint inhibitors OR ICI) AND (cardiotoxicity) |
| **OVID EMBASE**  (381 results) | (Immune checkpoint inhibitor OR ICI) AND (cardiotoxicity OR cardiac adverse events OR cardiac failure OR myocardial infarction OR pericardial effusion OR cardiac arrhythmia OR cardiac tamponade OR ventricular fibrillation OR myocarditis OR atrial fibrillation) AND (small cell lung cancer OR non small cell lung cancer OR lung cancer) |
| **Cochrane Library**  (619 results) | ''Lung Cancer'' AND ''Immune Checkpoint Inhibitor'' |

**Supplementary Table 2** Quality of Evidence by GRADE approach

| **Cardiac Adverse Events in Immune Checkpoint Inhibitor (ICI) Compared to Control for Lung Cancer** | | | | | |  |
| --- | --- | --- | --- | --- | --- | --- |
| Outcomes | **Anticipated absolute effects^*^** (95% CI) | | Relative effect (95% CI) | № of participants (studies) | Certainty of the evidence (GRADE) | |
|  | **Risk with Control** | **Risk with Immune Checkpoint Inhibitor** |  |  |  |  |
| Single ICI vs Chemotherapy | 4 per 1,000 | **9 per 1,000** (5 to 17) | **RR 2.15** (1.13 to 4.12) | 6929 (9 RCTs) | ⨁⨁◯◯ Low^a^ | |
| Single ICI plus Chemotherapy vs Chemotherapy | 21 per 1,000 | **29 per 1,000** (22 to 39) | **RR 1.38** (1.05 to 1.82) | 6870 (12 RCTs) | ⨁⨁⨁⨁ High | |
| Single ICI vs Dual ICI | 15 per 1,000 | **7 per 1,000** (2 to 26) | **RR 0.48** (0.13 to 1.80) | 1011 (4 RCTs) | ⨁⨁◯◯ Low^a,b^ | |
| ***The risk in the intervention group** (and its 95% confidence interval) is based on the assumed risk in the comparison group and the **relative effect** of the intervention (and its 95% CI).  **CI:** confidence interval; **RR:** risk ratio | | | | | |  |
| **GRADE Working Group grades of evidence** **High certainty:** we are very confident that the true effect lies close to that of the estimate of the effect. **Moderate certainty:** we are moderately confident in the effect estimate: the true effect is likely to be close to the estimate of the effect, but there is a possibility that it is substantially different. **Low certainty:** our confidence in the effect estimate is limited: the true effect may be substantially different from the estimate of the effect. **Very low certainty:** we have very little confidence in the effect estimate: the true effect is likely to be substantially different from the estimate of effect. | | | | | |  |

a. Imprecise as confidence interval included potential for important harm or benefit

b. some concern with performance bias and detection bias.

**Supplementary Figure 1** Flowchart of Study selection

**Supplementary Figure 2** Risk of bias graph: review authors' judgements about each risk of bias item presented as percentages across all included studies

**Supplementary Figure 3** Risk of bias summary: review authors' judgements about each risk of bias item for each included study

**Supplementary Figure 4** Funnel plot of incidence of any cardiac adverse events in lung cancer patients treated with immune checkpoint inhibitors


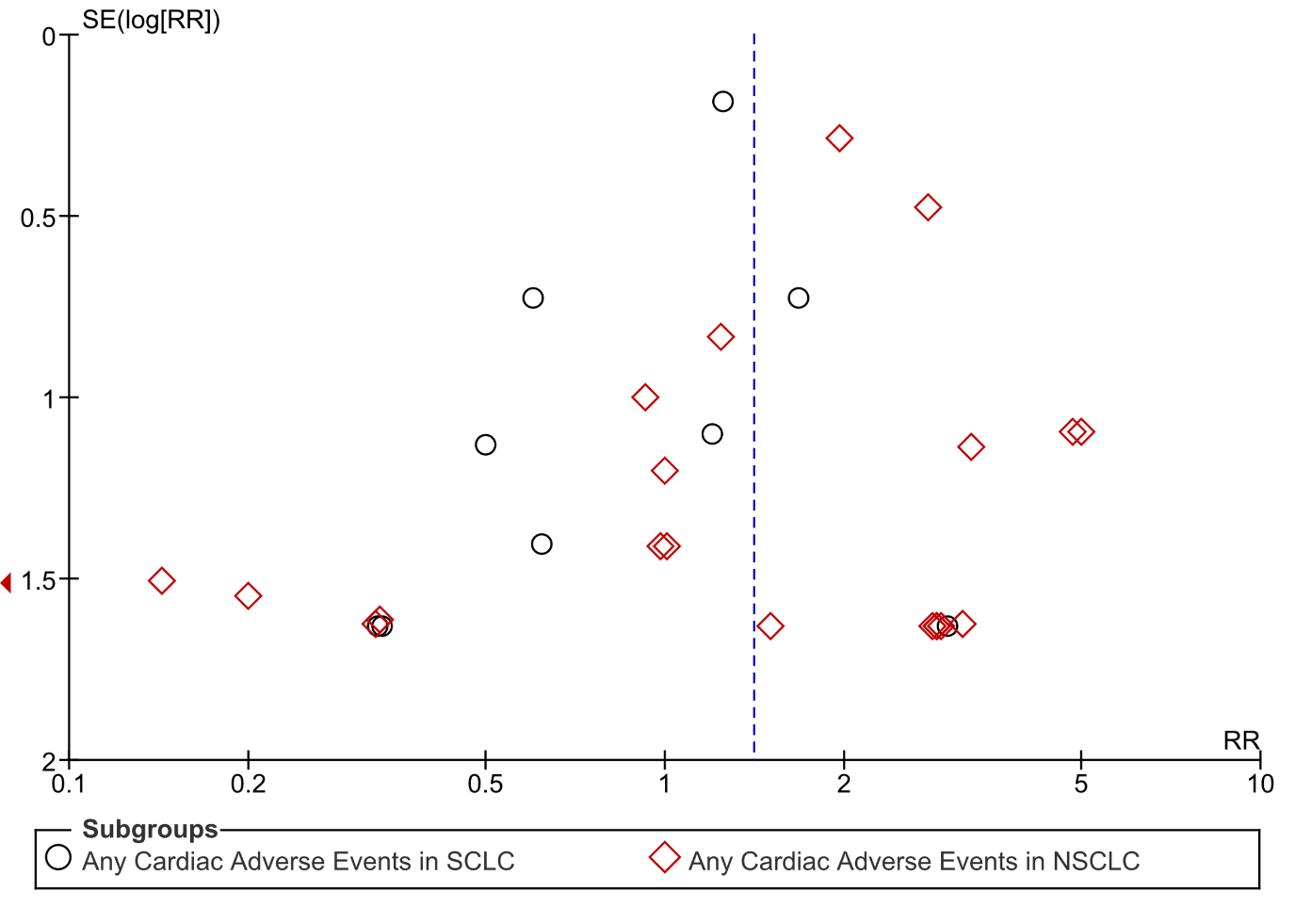


**Supplementary Figure 5** Forest plot of risk ratio of arrhythmia in lung cancer patients treated with immune checkpoint inhibitors vs control

**

**

**Supplementary Figure 6** Forest plot of risk ratio of myocardial infarction in lung cancer patients treated with immune checkpoint inhibitors vs control





**Supplementary Figure 7** Forest plot of risk ratio of atrial fibrillation in lung cancer patients treated with immune checkpoint inhibitors vs control

**

**

**Supplementary Figure 8** Forest plot of incidence of any cardiac adverse events in lung cancer patients treated with immune checkpoint inhibitors


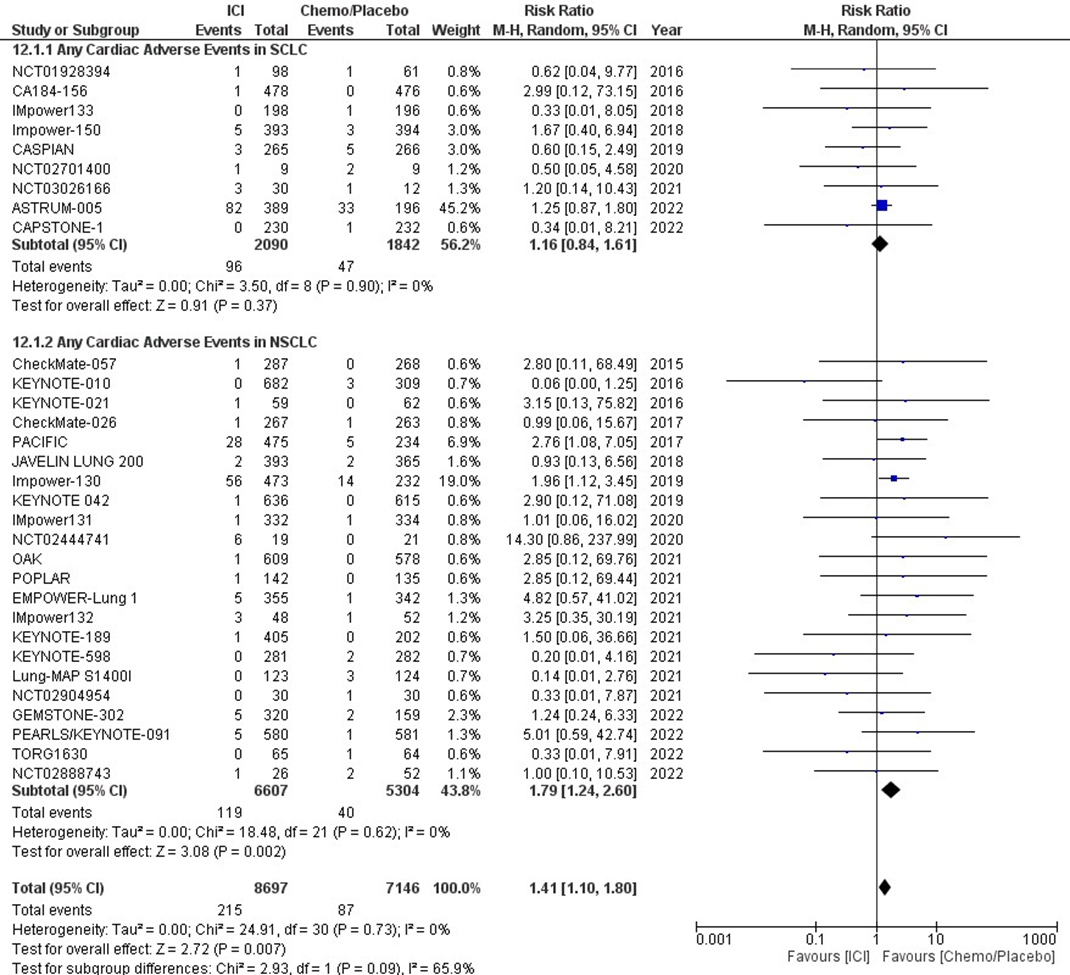

Supplement: Supplementary file 1 — Supplementary Material 1 [file 40959_2024_229_MOESM1_ESM.docx]
